# Faecal calprotectin is the biomarker that best distinguishes remission from different degrees of endoscopic activity in Crohn’s disease

**DOI:** 10.1186/s12876-020-1183-x

**Published:** 2020-02-13

**Authors:** Francisco Guilherme Cancela e Penna, Rodrigo Macedo Rosa, Pedro Ferrari Sales da Cunha, Stella Cristina Silva de Souza, Maria de Lourdes de Abreu Ferrari

**Affiliations:** 1 Instituto Alfa de Gastroenterologia, Hospital das Clínicas, Universidade Federal de Minas Gerais, Avenida Professor Alfredo Balena 110, second floor. Bairro: Santa Efigênia, Belo Horizonte, Minas Gerais CEP: 30130-100 Brazil; 2 Medical student, Faculdade de Medicina, Universidade Federal de Minas Gerais, Avenida Professor Alfredo Balena 190. Bairro: Santa Efigênia, Belo Horizonte, Minas Gerais CEP: 30130-100 Brazil; 3 Departamento de Clínica Médica, Faculdade de Medicina, Universidade Federal de Minas Gerais, Avenida Professor Alfredo Balena 190. Bairro: Santa Efigênia, Belo Horizonte, Minas Gerais CEP: 30130-100 Brazil

**Keywords:** Faecal calprotectin, Crohn’s disease, Simple endoscopic score for Crohn’s disease - SES-CD, C-reactive protein

## Abstract

**Background:**

Effective control of the inflammatory process in Crohn’s disease (CD) is reflected in intestinal mucosal healing. The performances of faecal calprotectin (fcal), clinical and serologic parameters in the inflammatory activity evaluation and their correlation to the simple endoscopic score (SES-CD) are the goals of this study.

**Methods:**

Patients with CD referred for ileocolonoscopy were prospectively included and distributed according to the degree of endoscopic inflammatory activity into remission, mild activity, and moderate to severe activity groups. The different degrees of endoscopic activity were correlated with the following indexes: Crohn’s disease activity index (CDAI), fCal, serum C-reactive protein (CRP), and haemogram. The control group comprised individuals without known intestinal disease who were referred for colorectal cancer screening.

**Results:**

Eighty colonoscopies were performed in patients with CD and 21 in the control group. The control group had a lower median fCal (59.7 mcg/g) than patients with CD (683 mcg/g, *p* < 0.001). A moderate Spearman correlation occurred between SES-CD and CRP (r = 0.525), fCal (*r* = 0.450), and CDAI (*r* = 0.407), while a weak correlation was found with the platelet count (*r* = 0.257). Only fCal distinguished patients in remission from those with mild activity (236.6 mcg/g × 654.9 mcg/g, *p* = 0.014) or moderate to severe activity (236.6 mcg/g × 1128 mcg/g, *p* < 0.001). An fCal cut-off of 155 mcg/g was sensitive (96%) and accurate (78%) for the diagnosis of endoscopic activity.

**Conclusions:**

fCal provides greater diagnostic accuracy than the other activity markers for endoscopic activity of patients with CD, moderate correlation to SES-CD, and a capacity to discriminate patients in remission from those with mild or moderate to severe activity.

## Background

Crohn’s disease (CD) is a recurrent disease with active and remissive periods, characterized by symptoms such as abdominal pain, fever, diarrhoea, and weight loss [1, 2]. Clinical progression is variable, and chronic inflammatory activity is responsible for structural damage of the intestine, even in patients who are asymptomatic or have prolonged clinical remission, representing approximately 10% of the studied samples [3, 4]. Since effective control of the inflammatory process is reflected in the healing of the intestinal mucosa and the healed mucosa is directly related to a lower recurrence, lower complication rates and the need for surgical treatment; it is necessary, for effective clinical practice, to use methods that safely measure intestinal inflammation [5–7]. An efficient therapeutic approach should aim to control tissue inflammation for mucosal healing and improve patient prognosis [8–12]. Frequent, objective, and regular evaluations of the inflammatory process are necessary to guide therapy [4, 13].

Ileocolonoscopy with biopsy of intestinal segments is the standard procedure for the evaluation of CD inflammatory activity [2, 14]. However, despite its usefulness, endoscopy entails a series of inconveniences: invasiveness, high cost, the need for anaesthesia, which presents its own risks; endoscopy is associated with potential bleeding of the mucosa and intestinal perforation [15, 16]. Furthermore, it is not always well tolerated by patients [8]. In developing countries, such as Brazil, it is also a somewhat inaccessible exam.

The ideal marker for the evaluation of CD inflammatory activity should be minimally invasive or noninvasive, low cost, easily disseminated, and accurate [16]. Unfortunately, none of the available markers possess all these traits. Clinical indexes, such as the Crohn’s disease activity index (CDAI), have a weak correlation with endoscopic activity and high subjectivity, both from a patient’s symptom report and a physician’s evaluation. The subjective aspect may correspond to 40% of the total score [17–19]. Serum C-reactive protein (CRP) may be altered during inflammation, infection, and tissue damage; has a short half-life; is easily accessible through blood sampling; and is useful in the sequential evaluation of inflammatory processes. However, CRP has a low specificity, since it can elevate during any inflammatory or infectious condition. For approximately 15% of the population, CRP remains unaltered even during an inflammatory process [20–22].

Hemogram alterations, such as anaemia, may be associated with CD inflammation. The anaemia has multiple origins, such as chronic disease anaemia, nutritional anaemia (deficiency of B12 vitamin, folic acid, and/or iron), or being secondary to medication. Increased platelet count is also an inflammatory activity marker, resulting in increased production of thrombopoietin due to interleukin-6 stimulation. Platelets participate in the perpetuation of the process since they release, in turn, pro-inflammatory mediators [9, 14, 23, 24].

Faecal markers, such as calprotectin and lactoferrin, are identified and quantified proteins in the faeces, and their measurement is useful since they are noninvasive, low cost, low risk, highly specific for intestinal inflammatory processes, and highly correlated to the endoscopic exam [22]. A review of the literature has shown a correlation between endoscopic scores and faecal calprotectin (fCal) [15, 25]. However, few studies have sought to establish cut-off points to define the presence and degrees of inflammatory activities. In the Brazilian population with CD, there are no studies comparing fCal with endoscopic scores. With the increased incidence and prevalence of the disease not only in this country but also throughout Latin America, evaluation of the usefulness of faecal calprotectin in patients in these countries is necessary [26, 27].

The present study sought to evaluate CD inflammatory activity using clinical and noninvasive laboratory markers (hemogram, C-reactive protein and faecal calprotectin) and correlate them to endoscopic findings, evaluated through the Simple Endoscopic Score for Crohn’s Disease – SES-CD [28]. The objective was to determine the ability of markers to discriminate between remission, mild, and moderate to severe activity during colonoscopy.

## Methods

### Sample

Between November 2011 and June 2016, 65 patients, 18 years of age or older and previously diagnosed with active or remission CD, referred to as ileocolonoscopy, were recruited from the patients seen in the Outpatient Unit for Intestinal Diseases or the Internal Medicine ward of the Instituto Alfa de Gastroenterologia of Hospital das Clínicas of the Federal University of Minas Gerais (IAG-HC/UFMG).

Of the 65 cases, 13 underwent ileocolonoscopy twice, and in one patient, the procedure was repeated on three distinct occasions during the study, for a total of 80 overall procedures. Exclusion criteria were refusal to undergo colonoscopy; inadequate intestinal preparation, impeding mucosal evaluation; incomplete exam due to technical difficulties; lack of stool sample for measurement of calprotectin; use of non-steroid anti-inflammatory drug; and the diagnosis of colorectal cancer, ostomies, or previous ileocolectomy.

The control group comprised 21 individuals with no history of intestinal disease, who underwent ileocolonoscopy for screening of colorectal cancer and presenting, at endoscopic exam, no polyps over 5 mm in diameter, diverticulitis, bleeding, or signs of intestinal mucosa inflammation.

The study was approved by the Ethics Committee of Research of the Federal University of Minas Gerais, filed as Parecer no. ETIC 00 70.0.203.000–11. All study participants provided written informed consent prior to enrollment.

### Clinical and laboratory evaluation

Recruited patients gave their written informed consent and, before colonoscopy, were interviewed and examined by the researcher. They filled in the card for CDAI score calculation, which is the clinical index for quantification of inflammatory activity. Blood and faecal samples were drawn for the measurement of the haemogram, serum C-reactive protein (CRP), and faecal calprotectin (fCal).

Faecal calprotectin was measured using the ELISA method with a commercial kit (BÜHLMANN Laboratories AG®) according to the manufacturer’s instructions. This method did not allow for measurements below 30 mcg/g or above 1800 mcg/g. Therefore, for statistical analysis, these values were applied to patients who presented values of ≤30 mcg/g or ≥ 1800 mcg/g, respectively.

### Endoscopic evaluation

An experienced practitioner, skilled in endoscopic scoring, performed all colonoscopies and was blind to the results of fCal, haemogram, CRP, and CDAI. The SES-CD score and the high definition Fujifilm scope (Fujifilm Co, Japan) were used to measure endoscopic disease activity.

The SES-CD scale proposed by the International Organization of Inflammatory Bowel Disease [29] and Koutroumpakis et al. [30] was used for dividing patients with CD into groups based on their endoscopic disease activity: group 1, endoscopic remission – SES-CD = 0–2; group 2, mild activity – SES-CD = 3–6; and group 3, moderate to severe activity – SES-CD ≥ 7. It was important to differentiate between patients in endoscopic remission and those with mild or moderate to severe activity since these various conditions lead to different therapeutic approaches.

### Statistics

A sample of at least 21 patients with CD for each of the three groups was determined through SES-CD, obtaining 80% statistical power in the detection of a 1 standard deviation (SD) difference in the average of the groups in relation to calprotectin. In the calculations, 1 SD was considered to be calprotectin 252.14 mcg/g, a value obtained in a study with a similar design [15], and the significance level was 0.05. Software Minitab 14 Release was used.

The Shapiro-Wilk test was used to verify the sample distribution. Variables did not present with a normal distribution, and values were presented as medians, with the interquartile range (P_25_ – P_75_). Fisher’s exact test and chi-square were used to verify the association of categorical variables between the groups. Spearman non-parametric test was used to evaluate the correlation of noninvasive markers for inflammatory activity with the endoscopic score.

The Mann-Whitney test was employed to analyse medians between two groups (control versus patients, patients in remission versus patients in activity). When the medians of the three groups of patients with CD were compared, the significance level was adjusted to 0.0167, which corresponds to Bonferroni’s correction, achieved through a significance level of 0.05 divided by the three groups. The Jonckheere-Terpstra or Kruskal-Wallis test was used to compare medians between the three groups.

Cut-off values for the noninvasive exams were obtained through the ROC curve, determined by the highest value for the sum of sensitivity and specificity, corresponding to the Youden Index. Five further values were selected for faecal calprotectin to ensure an emphasis on the sensitivity or specificity, as needed, to assess its usefulness as a marker. The area under the ROC curve was used to calculate the accuracy for each exam.

After checking the hypothesis of the association between the outcome variable (endoscopic activity assessed by SES-CD) and the possible explanatory variables (age, sex, disease location, age at diagnosis and CD behaviour) using Pearson’s chi-square test, Mann-Whitney and Fisher’s exact test, it was concluded that only the variable age had a *p*-value lower than the level of significance of 0.20. As only age was significant in the univariate analysis, but with a *p* value greater than 0.05, there was no adjustment of the multivariate model.

## Results

### Clinical characteristics

Most of the patients with CD were male (52.5%), and the median age was 34 years (P_25_ = 25 and P_75_ = 44). No significant differences were encountered between the three subgroups stratified according to endoscopic inflammatory activity and the Montreal classification [31, 32], as shown in Table 1. However, the control group, comprising 21 individuals without known intestinal disease, showed a predominance of female sex (76.2%) and a median age of 54 years (P_25_ = 47.5 and P_75_ = 62), a statistically significant difference in relation to the CD group (*p* < 0.001).

**Table 1:** Clinical characteristics of patients with Crohn’s disease and the control group

	Endoscopic Remission *n* = 27	Mild activity *n* = 21	Moderate to severe activity *n* = 32	*P* value
Gender:				
Male	11 (40.7%)	15 (71.4%)	16 (50.0%)	
Female	16 (59.3%)	6 (28.6%)	16 (50.0%)	
Age (P _25–75_)	40,0 (29.0–50.0)	41 (23.5–50.0)	32,5 (24–36.8)	0.102*
Montreal Classification	n (%)	n (%)	n (%)	
Age at diagnosis				
A_1_ (≤16 years)	3 (11.1)	1 (4.8)	4 (12.5)	0.149**
A_2_ (17–40)	16 (59.3)	15 (71.4)	25 (78.1)	
A_3_ (>40 years)	8 (29.6)	5 (23.8)	3 (9.4)	
Disease Phenotype				
B_1_	5 (18.5)	4 (19.0)	11 (34.4)	
B_1p_	5 (18.5)	6 (28.6)	9 (28.1)	
B_2_	8 (29.7)	5 (23.8)	9 (28.1)	0.342**
B_2p_	2 (7.4)	1 (4.8)	1 (3.1)	
B_3_	5 (18.5)	4 (19.0)	0 (0)	
B_3p_	2 (7.4)	1 (4.8)	2 (6.3)	
Location of disease				
L_1_	10 (37.0)	8 (38.1)	7 (21.9)	
L_2_	4 (14.8)	4 (19.0)	8 (25.0)	
L_3_	11 (40.8)	8 (38.1)	17 (53.1)	0.628**
L_1_+ L_4_	2 (7.4)	1 (4.8)	0 (0)	
Medication				
5-ASA	14 (51.9)	12 (57.1)	14 (43.8)	
Thiopurine	19 (70.3)	14 (66.7)	20 (62.5)	
Anti-TNF	6 (22.2)	0 (0)	11 (34.4)	
Antibiotics	4 (14.8)	9 (42.9)	2 (6.3)	
Prednisone	9 (33.3)	7 (33.3)	19 (59.4)	
Methotrexate	0 (0)	0 (0)	1 (3.1)	
Tacrolimus	0 (0)	0 (0)	1 (3.1)	

P_25_: 25thpercentile_;_ P_75_: 75thpercentile; Significant p value: *p* < 0.05. *Kruskal-Wallis Test; **Fisher Exact Test. Age at diagnosis: A1: below 16 years of age; A2: between 17 and 40 years of age; A3: above 40 years of age. Location: L1: ileum; L2: colon; L3: ileocolonic; L4: upper gastrointestinal tract. Behaviour: B1: inflammatory; B2: stenosing; B3: penetrating; p = perianal; Anti-TNF (infliximab, adalimumab); Antibiotics (metronidazole, ciprofloxacin)

After checking the hypothesis of association between the outcome variable (SES-CD) and the possible explanatory variables (age, sex, location of the disease, age at diagnosis, behaviour of CD and fCal) using the Pearson chi-square test, Kruskal-Wallis and Fisher’s exact test, the age, age at diagnosis and fCal were found to be associated with the SES-CD with a significance level of 0.20. When carrying out the ordinal logistic regression, only fCal was found to be significantly associated with the outcome.

### Correlation between endoscopic activity index and serologic and faecal markers for inflammation

The SES-CD of 80 colonoscopies was compared to CDAI (*r* = 0.407), CRP (*r* = 0.525), and fCal (r = 0.450); all presented with a moderate Spearman correlation, with statistical significance (*p* < 0.001). The platelet count showed a weak correlation (*r* = 0.257), although with statistical significance (*p* < 0.022). However, no significant correlation was found with the haemoglobin levels.

The fCal median for the control group was 59.7 mcg/g (P25 = 30 mcg/g and P_75_ = 224.2 mcg/g) and for the CD group was 683 mcg/g (P_25_ = 241.6 and P_75_ = 1531.8 mcg/g), which were significantly higher (*p* < 0.001). Higher levels of fCal were also observed when comparing the subgroups in disease remission (SES-CD ≤ 2: 236.6 mcg/g; P_25–75_: 90.7–810.5 mcg/g; *p* = 0.019) and those with endoscopic activity (SES-CD > 3: 1020.1 mcg; P_25–75_: 386–1800 mcg/g; *p* < 0.001) to the control group.

Noninvasive markers were evaluated for diagnostic accuracy of inflammatory activity. Median levels of the markers were compared between patients with endoscopic remission (SES-CD = 0–2) and those with endoscopic activity (SES-CD ≥ 3) (Table 2). The platelet count, CRP, and fCal could differentiate remission from endoscopic activity.

**Table 2:** Value of the medians and interquartile ranges (P_25_-P_75_) of non-invasive markers of patients in endoscopic remission (SES-CD = 0–2) and those with endoscopic activity (SES-CD ≥ 3)

	Endoscopic remission (P_25_ – P_75_) *n* = 27	Endoscopic activity (P_25_ – P_75_) *n* = 53	*P* value
CDAI	93.0 (39.0–152.0)	128.0 (64.0–228.0)	0.082
Hb (g/dl)	12.8 (11.5–14.2)	13.2 (12.4–14.5)	0.438
Platelets (× 10^3^/mm^3^)	286.0 (218.0–315.0)	313.0 (254.2–380.2)	0.041
CRP (mg/L)	5.0 (5.0–11.0)	16.6 (5.9–32.1)	<0.001
fCal (mcg/g)	236.6 (90.7–810.5)	1020.1 (387.0–1800.0)	<0.001

Values expressed in medians, P_25_ – P_75_: 25th and 75th percentiles.

Significant p value: *p* < 0.05. Test: Mann-Whitney test.

*CDAI* Crohn’s disease activity index, *Hb* Haemoglobin, *CRP* Serum C-reactive protein, *fCal* Faecal calprotectin

The medians of the CDAI score, haemoglobin, platelet count, CRP, and fCal were compared for SES-CD degrees to assess the performance of noninvasive markers to stratify the endoscopic activity levels (Table 3). Other than haemoglobin, the markers differed significantly in all the groups according to the endoscopic score. For the variables with significant statistical differences, further comparison between different endoscopic activity levels is shown in Table 4. The only marker that significantly differentiated between endoscopic remission and activity, both mild and moderate to severe, was fCal. CDAI distinguished between moderate to severe and mild activity and patients in remission. The CRP was useful to differentiate patients in remission from those with moderate to severe activity. The platelet count was not used for stratification of various levels of endoscopic activity (Table 4).

**Table 3:** Comparison of the values of the medians and interquartile ranges of non-invasive markers according to the degree of endoscopic activity (SES-CD) in 80 patients with Crohn’s disease

	SES-CD: 0–2 (P_25_ – P_75_) *n* = 27	SES-CD: 3–6 (P_25_ – P_75_) *n* = 21	SES-CD: ≥ 7 (P_25_ – P_75_) *n* = 32	*P* value
CDAI	93 (39–152)	73 (30–126)	194 (100.25–286.5)	0.002
Hb (g/dl)	12.8 (11.5–14.2)	13.5 (12.6–14.3)	13.05 (12.1–14.5)	0.677
Platelets (×10^3^/mm^3^)	286 (218–315)	310 (259–354)	338 (260–384)	0.035
CRP (mg/L)	5.0 (5–11.02)	12.25 (5–25.21)	18.15 (8.94–39.2)	<0.001
fCal (mcg/g)	236.6 (90.7–810.5)	654.9 (386–1549)	1128 (390.4–1800)	<0.001

Values expressed in medians, P_25_ – P_75_: 25^th^ and 75^th^ percentiles.

Significant *p* value: *p* < 0.05. Test: Test: Jonckheere-Terpstra.

*CDAI* Crohn’s disease activity index, *Hb* haemoglobin, *CRP* Serum C-reactive protein, *fCal* Faecal calprotectin

**Table 4:** Summary of results of the comparison between the medians of the non-invasive markers according to the degree of endoscopic activity (SES-CD)

	Remission X Mild activity	Remission X Moderate to severe activity	Mild activity X Moderate to severe activity
CDAI	*p* = 0.372	*p* = 0.001*	*p* = 0.002*
Platelets (× 10^3^/mm^3^)	*p* = 0.156	*p* = 0.045	*p* = 0.0504
CRP (mg/L)	*p* = 0.112	*p* < 0.001*	*p* = 0.074
fCal (mcg/g)	*p* = 0.014*	*p* < 0.001*	*p* = 0.157

* Significant *p* value: *p* < 0.0167 (Bonferroni’s correction). Test: Mann-Whitney test

*CDAI* Crohn’s disease activity index, *Hb* Haemoglobin, *CRP* Serum C-reactive protein, *fCal* Faecal calprotectin

### Determination of cut-off values for noninvasive exams

**Fig. 1:**
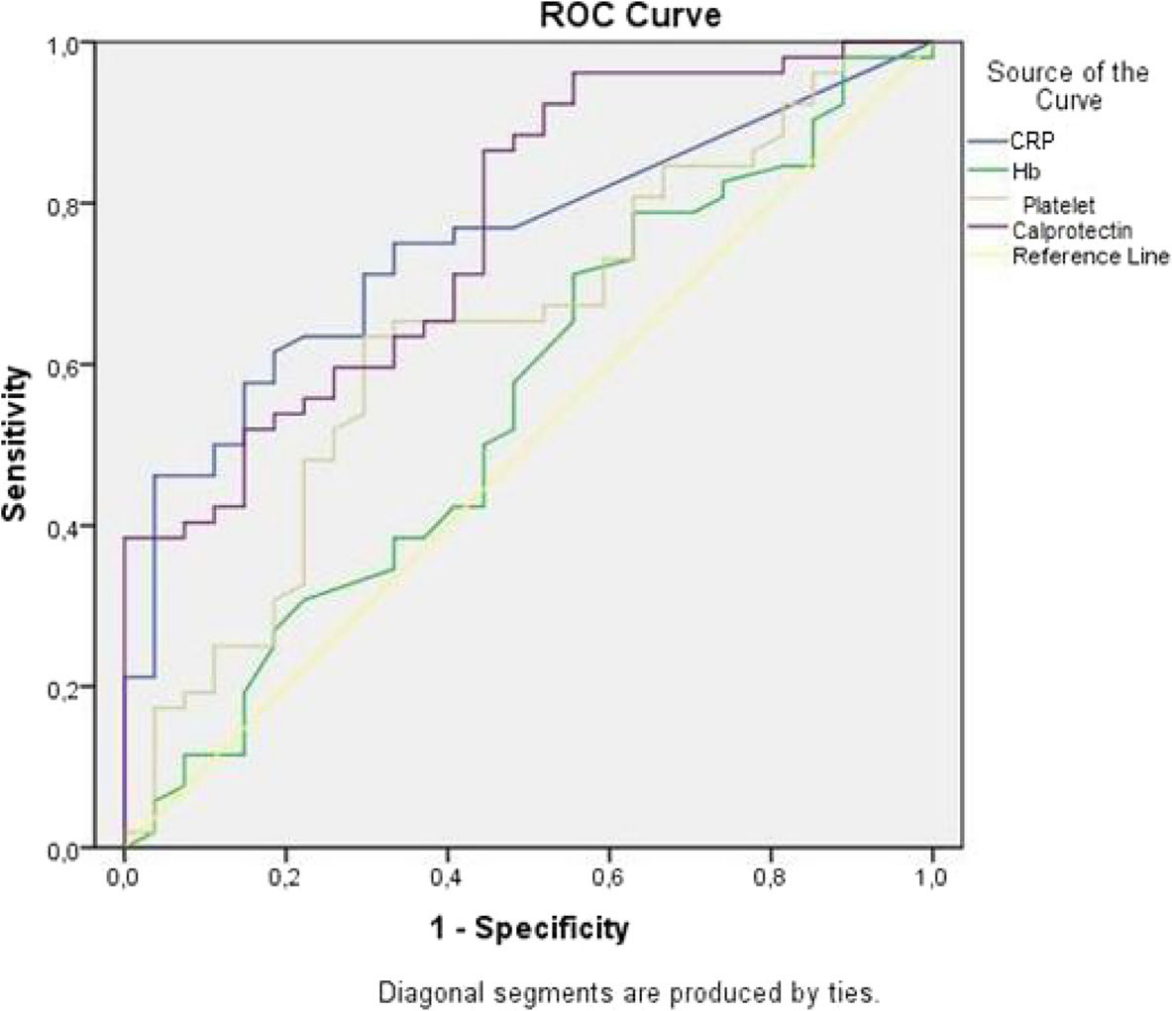
ROC curves for non-invasive markers. CRP: C-reactive protein; Hb: haemoglobin; Calprotectin: faecal calprotectin

Cut-off points for markers with a positive correlation to endoscopic alterations (platelet count, CRP, and fCal) were calculated through the ROC curve (Fig. 1). According to this curve, the accuracy and respective confidence intervals (CI) of the biomarkers for the diagnosis of inflammatory activity in patients with Crohn’s disease were 0.77 for faecal calprotectin (95% CI: 0.65–0.88; *p* < 0.001), 0.75 for CRP (95% CI: 0.64–0.86; *p* < 0.001), 0.64 for the platelet count (95% CI: 0.51–0.77; *p* = 0.041) and 0.55 for haemoglobin levels (95% CI, 0.42–0.69; *p* = 0.438).

Analysis was not performed for the haemoglobin cut-off points as it had no correlation to endoscopic activity or CDAI, since there are established cut-off points for remission and activity for this index in the literature.

The sensitivity, specificity, and likelihood ratios for positive and negative results of the different biomarkers for diagnosis of CD activity were calculated through cut-off points established with the ROC curve. There is no consensus in the literature regarding the ideal reference value for fCal for the diagnosis of intestinal inflammatory activity. Therefore, different cut-off points were selected to assess which had the best performance. The prevalence of inflammatory activity in the sample was applied to establish a positive predictive value, negative predictive value, and accuracy (Table 5).

**Table 5:** Sensitivity (Sens), specificity (Spec), positive predictive value (PPV), negative predictive value (NPV) and accuracy (A) for each biomarker and their respective cut-offs and 95% confidence intervals (95% CI) in the diagnosis of inflammatory activity in 80 patients with Crohn’s disease

	Sens	95% CI	Spec	95% CI	PPV	95% CI	NPV	95% CI	A	95% CI
fCal≥155 mcg/g	0.96	0.91–1.0	0.44	0.27–0.61	0.77	0.68–0.86	0.86	0.68–1.0	0.78	0.70–0.86
fCal≥199 mcg/g	0.90	0.83–0.97	0.48	0.31–0.65	0.77	0.68–0.86	0.72	0.53–0.90	0.76	0.67–0.84
fCal≥246 mcg/g	0.89	0.82–0.97	0.52	0.35–0.69	0.78	0.69–0.88	0.70	0.53–0.89	0.76	0.68–0.85
fCal≥273 mcg/g	0.87	0.79–0.95	0.56	0.39–0.73	0.79	0.70–0.88	0.68	0.52–0.86	0.76	0.68–0.85
fCal≥1009 mcg/g	0.52	0.40–0.64	0.85	0.73–0.97	0.87	0.77–0.98	0.48	0.35–0.60	0.63	0.54–0.85
fCal≥1473 mcg/g	0.40	0.28–0.52	0.93	0.84–1.0	0.91	0.82–1.0	0.44	0.33–0.56	0.58	0.48–0.68
PCR ≥ 6.7 mg/L	0.75	0.65–0.85	0.67	0.51–0.83	0.81	0.72–0.91	0.58	0.43–0.73	0.72	0.64–0.81
Plaq≥ 292,500/mm^3^	0.65	0.54–0.74	0.67	0.51–0.83	0.79	0.69–0.90	0.5	0.35–0.64	0.66	0.56–0.75

*fCal* Faecal calprotectin, *CRP* serum C-reactive protein, *Platelets* Platelet count, *95% CI* 95% confidence interval, *Sens* Sensitivity, *Spec* Specificity, *PPV* Positive predictive value, *NPV* Negative predictive value, *A* Accuracy

The same cut-off values for biomarkers were used to assess the likelihood ratios for positive and negative results, posttest odds ratio, and posttest likelihood of CD activity, as shown in Table 6.

**Table 6:** Likelihood ratio for positive result (LR+), likelihood ratio for negative result (LR-) and its inverse (1/LR-), posttest odds ratio (posttest OR), and probability following tests (posttest P) of Crohn’s disease activity for each noninvasive marker exam and their respective cut-offs

	LR+	LR-	1/LR-	Posttest OR	Posttest P
fCal≥155 mcg/g	1.73	0.08	11.69	3.36	0.77
fCal≥199 mcg/g	1.74	0.20	5.01	3.38	0.77
fCal≥246 mcg/g	1.83	0.22	4.49	3.56	0.78
fCal≥273 mcg/g	1.95	0.24	4.12	3.77	0.79
fCal≥1009 mcg/g	3.50	0.56	1.77	6.80	0.87
fCal≥ 1473 mcg/g	5.45	0.64	1.55	10.58	0.91
CRP ≥ 6.7 mg/L	2.25	0.38	2.66	4.36	0.81
Pla ≥ 292,500/mm^3^	1.96	0.51	1.92	3.80	0.79

*fCal* Faecal calprotectin, *CRP* Serum C-reactive protein, *Pla* Platelet count, *LR+* Likelihood ratio for positive result, *LR-* Likelihood ratio for negative result, *1/LR-* Likelihood ratio in patients in remission, *OR* odds ratio, *Posttest P* Posttest probability of CD activity

## Discussion

The diagnosis and follow-up of patients with inflammatory intestinal disease is frequently complex and includes a combination of clinical, laboratory, endoscopic, histopathologic, and radiologic aspects [14, 33]. Periods of inflammatory activity, particularly for CD, are frequently underestimated or recognition is delayed due to a lack of specificity or the invasiveness of available procedures [17, 22, 34]. Biomarkers that determine type, severity, prognosis, and therapeutic response are clinically relevant and have a direct impact on therapeutic decisions and, consequently, on patient prognosis. Researchers in the field are in constant search of an ideal biomarker.

Groups with CD and different levels of endoscopic inflammatory activity were similar for sex, age, and disease phenotype. Significant differences were observed in relation to the control group, which was older than the CD group. This was due to the profile of recruited individuals, since their referral to ileocolonoscopy was for colorectal cancer screening.

The markers with the best correlation to SES-CD were CRP (*r* = 0.525), fCal (*r* = 0.450), CDAI (*r* = 0.407), and platelet count (*r* = 0.257). These results are similar to those reported by Sipponenet al [25], .who evaluated 87 patients with CD and found the following correlations to SES-CD: fCal (*r* = 0.662), CRP (*r* = 0.522), CDAI (*r* = 0.346), and platelet count (*r* = 0.290). The results of Schoepfer et al. [15] and Lin et al. [35] corroborated these findings.

Haemoglobin showed no correlation to endoscopic activity, as also described by Sipponenet al [25]. CD’s anaemia is multifactorial by nature and reflects not only enteric bleeding but also chronic inflammatory activity, vitamin and mineral deficiency, and adverse reactions with medication; this may account for the lack of correlation to intestinal injury [23].

Faecal calprotectin has been shown to be useful in differentiating patients with CD from individuals without inflammatory bowel disease, a finding that is similar to the literature data [15, 36–39]. A significant difference was observed even when CD in remission was compared to the control group.

Mucosal healing is the main goal for inflammatory bowel disease treatment, as it is known to be directly related to decreased permanent structural damage to the gastrointestinal tract [5, 7, 34]. Therefore, the identification of markers that can safely distinguish between remission and endoscopic activity, including mild activity, is important. Colonoscopy is the standard method; however, it is limited by its invasiveness. In this study, platelet count, CRP, and fCal were shown to be useful in differentiating patients in remission from those with endoscopic activity. Of these, fCal was the only one that distinguished remission (SES-CD ≤ 2) from mild or moderate to severe activity. This result, particularly the distinction between remission and mild endoscopic activity, is an important contribution to the clinical approach since it can guide early treatment adjustments, with positive repercussions for long-term outcomes of the disease [10, 40, 41]. Similar results were described in studies that took place in Europe [15, 25], North America [42], and Asia [35]; however, there are no reports of similar assessments in the Latin American population.

The ROC curve identified cut-off reference values for biomarkers with the greatest sensitivity and specificity for the diagnosis of inflammatory activity. Among the studied markers, fCal showed the best diagnostic accuracy. For CRP, the cut-off of 6.7 mg/L showed satisfactory diagnostic accuracy, with more sensitivity in relation to specificity. This result differs from the findings of Schoepfer et al. [15], in which sensitivity and specificity had lower values. The platelet count had intermediate values both for sensitivity and specificity and was shown to be useful as an adjunct marker in the identification of CD intestinal inflammation.

Analysis of the cut-off values for fCal showed that 273 mcg/g had the best sensitivity and specificity, as shown by the Youden Index. There is no consensus in the literature regarding the best value for the characterisation of inflammatory activity in CD. To distinguish different values used for the diagnosis of inflammatory activity, values similar to previous reports in the literature and with a high Youden index were chosen: 155 mcg/g, 199 mcg/g, and 246 mcg/g. Two other fCal values were selected, 1009 mcg and 1473 mcg/g, to evaluate the performance of a value with greater specificity, since the other values prioritised sensitivity.

In evaluating positive and negative predictor values and the accuracy of selected cut-offs, 155 mcg/g was the best result. This value had excellent sensitivity (96%) and a 78% diagnostic accuracy, demonstrating the potential for usefulness in clinical practice since it is able to identify almost all patients with endoscopic inflammatory activity. Based on an analysis of the likelihood ratios, values lower than 155 mcg/g are eleven times more likely in patients in disease remission, which may support follow-up without a need for colonoscopy.

Many studies suggest cut-off points of 200 mcg/g or 250 mcg/g for the diagnosis of inflammatory activity in CD, which are similar to the values 199 mcg/g or 243 mcg/g found in this study [16, 22]. Furthermore, some studies consider values of up to 280 mcg/g [43, 44] for the diagnosis of CD activity, which is close to the value 273 mcg/g found in this sample. In the studied sample, such values showed decreased sensitivity when compared to the cut-off of 155 mcg/g, without a significant increase in specificity or positive predictor value. However, accuracy remained similar for all these values.

High specificity for the diagnosis of inflammatory activity was found for cut-off values of 1009 mcg/g and especially 1473 mcg/g, with a posttest likelihood of 91% for the latter and 87% for the former, when the result was positive. However, concurrent with the increased specificity, there is a loss of sensitivity. A high cut-off value for fCal was also studied by Lin et al. [35], with a value of 918 mcg obtaining 100% specificity and 50% sensitivity.

Based on these data, the cut-off value of 155 mcg/g for fCal was shown to be useful to evaluate the treatment goal of mucosal healing in CD, since this cut-off value privileges sensitivity and has a high negative predictive value, both of which are important traits for the exclusion of intestinal mucosal injury.

This study had potential limitations. The first was the option of not performing serial measurements of calprotectin, which could increase the accuracy of this test. However, this was also a strategy adopted by other authors in different studies. Second, only one kit of calprotectin was used, which, although already validated, does not allow extrapolation to other methods of dosing this marker. Third, the study of the small intestine was not performed through enterography, which could allow more reliable conclusions about the inflammatory activity. However, all patients included in this study had ileal or ileocolonic involvement, allowing us to infer that ileocolonoscopy could be used to diagnose inflammatory activity in this sample. Finally, the number of patients in the endoscopic remission group, although estimated before the start of the study, was responsible for the large confidence intervals found in the specificity values.

## Conclusions

The study permitted the following conclusions: fCal, CRP, CDAI, and platelet count showed correlation to endoscopic activity; fCal had a greater diagnostic capacity since it allowed for a differential diagnosis between endoscopic remission and mild or moderate to severe activity; the cut-off level for fCal of 155 mcg/g appears to be ideal for the noninvasive assessment of inflammatory activity as it presents with good sensitivity (96%), a diagnostic accuracy of endoscopic activity of 78%, and a significant negative predictor value; values above 1009 mcg/g and, specifically those over 1473 mcg/g, are indicative of intestinal inflammatory activity in CD, and the value of 1128 mcg / g correlates with moderate to severe endoscopic activity.

## Supplementary information

**Additional file 1:** Supplementary material: Clincial, laboratorial and endoscopic data.

## Data Availability

All data generated or analysed during this study are included in this published article. The datasets generated and analysed during the current study are available from the corresponding author by email cancelapenna@gmail.com on reasonable request.

## Abbreviations

A1: Below 16 years of age
A2: Between 17 and 40 years of age
A3: Above 40 years of age
B1: Inflammatory
B2: Stenosing
B3: Penetrating
CD: Crohn’s disease
CDAI: Crohn’s disease activity index
CRP: C-reactive protein
fCal: faecal calprotectin
IAG-HC/UFMG: Instituto Alfa de Gastroenterologia of Hospital das Clínicas of the Federal University of Minas Gerais
p: Perianal
P_25_ – P_75_: Interquartile range
SD: Standard deviation
SES-CD: Simple endoscopic score for Crohn’s disease
